# Meiotic abnormalities affect genetic constitution and pollen viability in dicots from Indian cold deserts

**DOI:** 10.1186/s12870-018-1596-7

**Published:** 2019-01-07

**Authors:** Dalvir Kaur, V. K. Singhal

**Affiliations:** 1 Department of Botany, Patel Memorial National College, Rajpura affiliated to Punjabi University, Patiala, Punjab India; 2 Department of Botany, Punjabi University, Patiala, Punjab India

**Keywords:** Meiosis, Meiotic abnormalities, Pollen grains, Aneuploids, Polyploids, Sexual reproduction

## Abstract

**Background:**

Meiotic abnormalities lead to morphological and genetic variations which caused not only to evolution but also intraspecific reproductive barriers. During present study of detailed meiotic course in dicotyledonous plants sampled from Indian cold deserts, various meiotic abnormalities have been detected. For this, the plant materials fixed in Carnoy’s fixative and studied detailed meiotic course by standard squash method in 1% acetocarmine.

**Results:**

Meiotic abnormalities have been presently detected in 71 species which include multiple associations in diploids (*Achillea millefolium* L.), multivalents and univalents in polyploids (4 species), cytomixis (40 species), chromosome stickiness (20 species), nonsynchronous disjunction of bivalents (32 species), interbivalent connections (15 species), synaptic mutants (2 species), syncyte meiocytes (2 species), abnormal spindles (7 species), and fusion of pollen grains (1 species), laggards and chromatin bridges, hypo-, hyperploid PMCs, monads, dyads, triads, tetrads with micronuclei and polyads.

**Conclusions:**

Consequently, variable sized apparently fertile pollen grains and considerable amount of sterile pollen grains are resulted as end products which lead to different genetic constitution (aneuploids and polyploids) and curtailed sexual reproductive success in these species.

## Background

Meiosis, [1] a critical process has a key role in the reproduction and life cycle of flowering plants involves homologous chromosomes pair, synapsis, recombination and segregation that reduces the chromosome number by half and ensures the operation of Mendel’s law of heredity [2]. The normal and harmonious course of meiosis in pollen mother cells including regular bivalent formation and normal cytokinesis ensures 100% pollen viability [3]. Any abnormality in course of meiosis causes the formation of sterile gametes and low percentage of pollen viability [4]. An array of genes is known to involve in each and every step of meiotic process [3, 5–7]. Further alongwith genic factors, various environmental factors determine the harmony of this process [8]. To investigate the evolutionary trends occurring in the species, a detailed meiotic analysis could be a strong parameter which includes the nature of chromosomes pairing, chromosome behaviour during segregation and microsporad formation [9]. Formation of genetically different gametes due to abnormal meiotic processes could lead to effective reproductive barriers between the species [10]. During the evaluation of meiotic behavior in the dicot plants sampled from Kinnaur district of Himachal Pradesh (India) which is known for its rugged mountains, cold deserts, high altitudes, harsh climatic conditions and remote district in India, 71 species showed various meiotic abnormalities which curtailed the considerable amount of pollen fertility and led to the production of heterogeneous sized fertile pollen grains.

## Results

In our present studies total 71 taxa (22 polyploids and 49 diploids) showed abnormal meiotic abnormalities (Table 1) which include chromosomal multiple associations in diploids and polyploids, chromatin stickiness, deviant pairing and disjunction of bivalents, cytomixis, amalgamated meiocytes, abnormal spindle, laggards and chromatin bridges, anomalous sporads, and united pollen grains. Inconstant sized fertile gametes and significant pollen sterility are the final outcome of all these abnormalities in this present study.

**Table 1:** A list of cytologically investigated species with locality, accession number, meiotic chromosome number, meiotic abnormalities and pollen grains

	Taxon	Locality (altitude in metre)	Accession number (PUN^a^)	MCN (Ploidy level)	Meiotic irregularities									Pollen grains		
					C	CS	U	M	AM	ND	L	CB	AbM	PS	HSPG	FPG
1.	FAMILY: RANUNCULACEAE															
	*Anemone rivularis* Buch.-Ham. ex DC.	Kalpa, 2760	50898	8 (2x)	+	+	-	-	-	-	+	+	+	+	+	-
2.	*Aquilegia fragrans* Benth*.*	Chittkul, 3450	53994	7 (2x)	+	-	-	-	-	-	-	-	-	-	-	-
3.	*Clematis grata* Wall.	Bhabnagar, 1900	50905	8 (2x)	+	+	-	-	-	-	+	+	+	+	-	-
4.	*C. graveolens* Lindl.	Thangi, 2700	53858	8 (2x)	+	+	-	-	-	+	+	+	+	+	+	+
5.	*C. orientalis* L. var. *acutifolia* Hook. f. *et* Thoms.	Nako, 3660	49994	16 (4x)	+	+	-	-	-	+	+	+	+	+	+	-
6.	*Delphinium roylei* Munz	Sangla, 2680	53998	8 (2x)	-	-	-	-	-	+	-	-	-	-	-	-
7.	*Ranunculus laetus* Wall. ex Royle	Sangla, 2680	50946	14 (4x)	+	+	-	-	-	+	+	+	+	+	+	-
8.	*R. sceleratus* L.	Nichar, 2150	54006	16 (4x)	-	+	-	-	-	+	+	+	+	+	+	-
9.	*Thalictrum cultratum* Wall.	Nichar, 2150	53847	21 (4x)	+	-	-	-	-	+	+	+	+	+	+	-
10.	*T. foetidum* L.	Sangla, 2680	50947	21 (6x)	+	-	-	-	-	-	+	+	+	+	+	-
11.	*T. minus* L.	Chittkul, 3450	53887	7 (2x)	+	-	-	-	-	-	-	-	-	-	-	-
12.	FAMILY: BERBERIDACEAE															
	*Berberis kunwarensis* Royle	Sangla, 2680	54025	14 (2x)	-	+	-	-	-	+	-	+	-	-	-	-
13.	FAMILY: PAPAVERACEAE															
	*Papaver dubium* L.	Sangla, 2680	50952	14 (4x)	-	-	-	-	-	+	+	+	+	+	+	-
14.	FAMILY: CARYOPHYLLACEAE															
	*Dianthus angulatus* Royle ex Benth.	Sangla, 2680	50486	15 (2x)	+	-	+	-	+	-	+	-	+	+	+	-
15.	*Myosoton aquaticum* (L.) Moench	Nichar, 2150	53734	14 (2x)	-	+	-	-	-	-	-	-	-	-	-	-
16.	*Silene edgeworthii* Bocquet	Sangla, 2680	50961	12 (2x)	+	-	-	-	-	-	-	-	+	+	+	-
17.	*Spergularia diandra* (Guss.) Heldr. & Sart.	Chango, 3050	53741	18 (4x)	+	+	-	-	-	-	+	-	+	+	+	-
18.	FAMILY GERANIACEAE															
	*Geranium pratense* L.	Chittkul, 3450	50924	28(4x)	-	+	-	+	-	-	-	-	+	+	+	-
19.	FAMILY: BALSAMINACEAE															
	*Impatiens brachycentra* Kar. *et* Kir.	Reckong Peo, 2670	50888	7 (2x)	-	-	-	-	-	+	-	-	-	-	-	-
20.	FAMILY: PAPILIONACEAE															
	*Astragalus grahamianus* Royle ex Benth.	Rakchham, 3115	53673	8 (2x)	+	-	-	-	-	-	+	-	+	+	-	-
21.	*A. graveolens* Buch.-Ham. ex Benth.	Sangla, 2680	50900	8 (2x)	+	+	-	-	-	-	+	+	+	+	+	-
22.	*Colutea nepalensis* Sims	Khab, 2800	53684	8 (2x)	-	-	-	-	-	-	+		+	+	-	-
23.	*Indigofera heterantha* Wall. ex Brandis	Rakchham, 3115	50912	24 (6x)	+	-	-	-	-	-	-	-	-	+	-	-
24.	*Lotus corniculatus* L.	Chitkul, 3450	53690	6 (2x)	+	-	-	-	-	-	+	+	+	+	-	-
25.	*Medicago falcata* L.	Thangi, 2700	53695	8 (2x)	+	-	-	-	-	-	-	-	+	+	-	-
26.	*Melilotus alba* Lamk.	Thangi, 2700	53699	8 (2x)	+	-	-	-	-	-	-	-	+	+	-	-
27.	*Trifolium repens* L.	Sangla, 2680	50916	16 (4x)	+	-	-	-	-	-	-	-	+	+	-	-
28.	*Trigonella emodi* Benth.	Chitkul, 3450	53713	8 (2x)	+	-	-	-	-	-	-	-	+	+	+	-
29.	*T. pubescens* Edgew. ex Baker	Rakchham, 3115	53716	8 (2x)	+	-	-	-	-	+	+	+	+	+	+	-
30.	*Vicia pallida* Turcz.	Palingi, 1900	53708	12 (4x)	+	+	-	-	-	+	+	+	+	+	+	-
31.	*V. rigidula* Royle	Kalpa, 2760	50919	12 (4x)	+	+	-	-	-	+	+	+	+	+	+	-
32.	*V. sativa* L.	Kuppa, 2600	53709	6 (2x)	-	-	-	-	-	+	-	+	-	-	-	-
33.	*V. tenera* Grah.	Sangla, 2680	51952	7 (2x)	-	-	-	-	-	+	+	+	+	+	-	-
34.	FAMILY: ROSACEAE															
	*Fragaria nubicola* (Hook.) Lindl. ex Lacaita	Chittkul, 3450	53797	7 (2x)	+	-	-	-	-	-	-	-	+	-	-	-
35.	*Potentilla atrisanguinea* Lodd. var. *atrisanguinea*	Chittkul, 3450	53840	28 (8x)	+	-	-	-	-	-	-	-	+	+	+	-
36.	FAMILY: APIACEAE															
	*Chaerophyllum acuminatum* Lindl.	Chittkul, 3450	53824	11(2x)	+	-	-	-	-	-	+	+	+	+	-	-
37.	*C. villosum* Wall.	Kuppa, 2600	53826	11 (2x)	-	-	-	-	-	+	+	-	+	+	-	-
38.	*Eriocycla caespitosa* (Edgew.) H. Wolff	Sangla, 2680	53823	11 (2x)	+	-	-	-	-	-	+	-	+	+	-	-
39.	*Heracleum candicans* Wall. ex DC.	Rakchham, 3115	53828	11 (2x)	+	+	-	-	-	-	+	+	+	+	+	-
40.	*SUB CLASS - GAMOPETALAE*															
	FAMILY: ASTERACEAE															
	*Achillea millefolium* L.	Chittkul, 3450	53873	9 (2x)	-	-	-	-	-	-	+	+	+	+	-	-
41.	*Anaphalis virgata*	Chittkul, 3450	53876	14 (4x)	-	-	-	-	-	+	-	-	-	-	-	-
42.	*Aster molliusculus* Wall.	Pooh, 2840	53914	9 (2x)	-	-	-	-	-	+	-	-	-	-	-	-
43.	*Carduus nutans* L.	Kamru, 2650	53893	20 (4x)	-	-	-	+	-	-	-	-	-	-	-	-
44.	*Cosmos bipinnatus* Cav.	Pangi, 2790	53892	12 (2x)	-	-	-	-	-	+	-	-	-	-	-	-
45.	*Crepis multicaulis* Ledeb.	Kothi, 2670	53897	5 (2x)	+	-	-	-	-	+	-	+	+	+	-	-
46.	*C. sancta* (L.) Babcock	Kuppa, 2600	53908	5 (2x)	-	-	-	-	-	+	-	+	-	-	-	-
47.	*Erigeron acer* L.	Sangla, 2680	53915	9 (2x)	-	-	-	-	-	+	+	-	+	+	-	-
48.	*E. annuus* (L.) Pers.	Sangla, 2680	53917	27 (3x)	-	+	+	-	-	-	+	+	+	+	+	-
49.	*Inula cappa* DC.	Palingi, 1900	53937	10 (2x)	-	-	-	-	-	+	-	+	+	+	+	-
50.	*I. cuspidata* C.B. Clarke	Tapri, 1680	52513	10 (2x)	-	-	+	-	+	+	+	+	+	+	+	-
51.	*Lactuca orientalis* (Boiss.) Boiss.	Nako, 3660	53948	9 (2x)	-	-	-	-	-	+	+	-	+	+	-	-
52.	*Saussurea albescens* Hook. f. *et*. Thoms.	Ropa, 3000	53969	17 (2x)	-	-	-	-	-	-	+	-	-	+	-	-
53.	*Senecio krascheninnicovi* Schischk	Nako, 3660	53961	10 (4x)	+	-	-	-	-	+	+	-	+	+	+	-
54.	*Taraxacum officinale* F. H. Wigg.	Ropa, 3000	55765	16 (4x)	-	-	-	+	-	-	+	+	+	+	+	-
55.	*Youngia glauca* Edgew.	Nichar, 2150	53896	8 (2x)	-	-	-	-	-	+	+	-	-	+	-	-
56.	FAMILY: BORAGINACEAE															
	*Cynoglossum zeylanicum* (Wall.) Thunb. ex Lehm.	Moorang, 2590	53835	12 (2x)	+	-	-	-	-	-	+	+	+	+	-	-
57.	FAMILY: CUSCUTACEAE															
	*Cuscuta reflexa* Roxb.	Ponda, 1980	54073	16 (4x)	-	+	-	-	-	+	+	+	-	-	-	-
58.	FAMILY: SOLANACEAE															
	*Datura stramonium* L.	Kalpa, 2760	50968	12 (2x)	-	-	-	-	-	-	+	+	-	+	-	-
59.	*Hyoscyamus niger* L.	Nako, 3660	53780	17 (2x)	-	-	-	-	-	-	-	+	-	-	-	-
60.	*Nicotiana tabacum* L.	Tapri, 1680	53784	24 (4x)	+	+	-	-	-	-	-	+	+	+	-	-
61.	*Physalis minima* L.	Ponda, 1980	53785	24 (4x)	-	-	-	-	-	+	+	-	+	+	-	-
62.	FAMILY: SCROPHULARIACEAE															
	*Leptorhabdos benthamiana* Walp.	Kalpa, 2760	54112	7 (2x)	+	-	-	-	-	+	+	+	+	+	+	-
63.	*Pedicularis bicornuta* Klotzsch	Sangla, 2680	50936	8 (2x)	+	+	-	-	-	+	+	+	+	+	+	-
64.	FAMILY: LAMIACEAE															
	*Calamintha clinopodium* Benth.	Chittkul, 3450	54125	10 (2x)	+	-	-	-	-	-	+	-	-	+	-	-
65.	*Mentha longifolia* (L.) Huds.	Pooh, 2840	54139	12 (2x)	+	-	-	-	-	-	-	+	-	-	-	-
66.	*Nepeta erecta* (Royle ex Benth.) Benth.	Kuppa, 2600	53606	9 (2x)	+	-	-	-	-	-	-	+	+	+	-	-
67.	*Salvia nubicola* Wall. ex Sweet	Sangla, 2680	50930	8 (2x)	+	-	-	-	-	+	+	+	+	+	-	-
68.	*Thymus linearis* Benth.	Kuppa, 2600	53631	13 (2x)	+	-	-	-	-	-	+	-	+	+	+	-
69.	*SUB CLASS - MONOCHLAMYDAE*															
	FAMILY: PHYTOLACCACEAE															
	*Phytolacca acinosa* Roxb.	Sangla, 2680	54074	36 (8x)	-	-	-	-	-	-	+	+	-	+	-	-
70.	FAMILY: POLYGONACEAE *Rumex hastatus* D. Don	Kothi, 2670	50895	9 (2x)	-	-	-	-	-	-	-	+	-	-	-	-
71.	FAMILY: ELAEAGNACEAE															
	*Hippophae rhamnoides* L.	Kalpa, 2760	49377	9 (2x)	+	+	-	-	-	+	+	+	+	+	+	-

Symbol + (presence) and – (absence) of meiotic irregularities

^a^ Code of Herbarium maintained by the Department of Botany, Punjabi University, Patiala, India as per “Index Herbariorum” by Holmgren and Holmgren (1998)

*MCN* Meiotic Chromosome Number; *C* Cytomixis; *CS* Chromosome Stickiness; *U* Univalents; *M* Multivalents; *AM* Asynaptic Mutant; *ND* Nonsynchronous Disjunction; *L* Laggard; *CB* Chromatin Bridge; *AbM* Abnormal Microspore; *PS* Pollen Sterility; *HSPG* Heterogenous Sized Pollen Grains; *FPG* Fused Pollen Grains

### Chromosomal multiple association in diploids

**Fig. 1:**
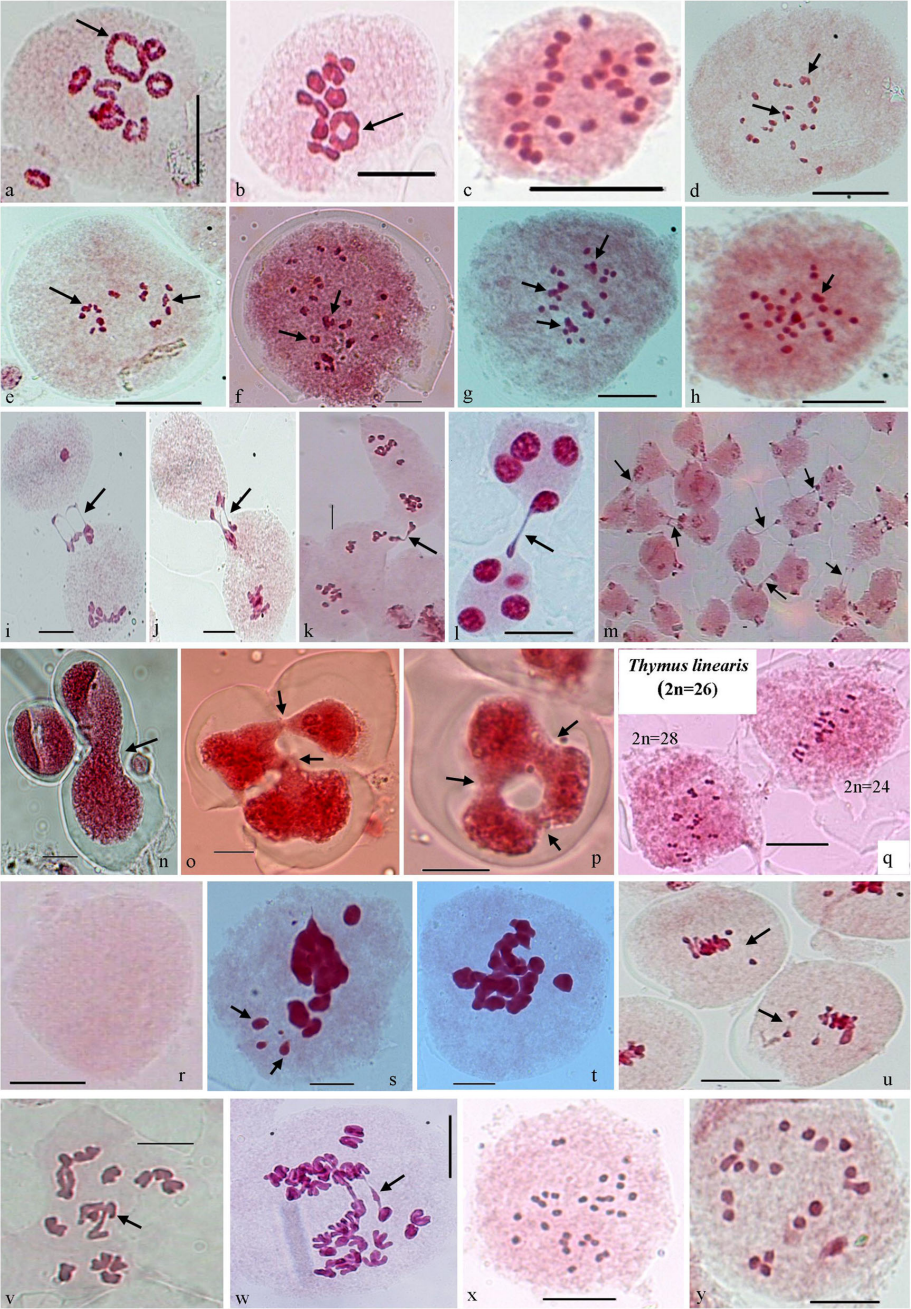
Various Meiotic deformities during meiosis.  **a**) *Achillea millefolium* (2n=18); A PMC with 7_II_+1_IV_ (typical ring, arrowed) at diakinesis. **b**) *A. millefolium* (2n=18); PMC with 7_II_+1_IV_ (typical ring, arrowed) at M-I. **c**) *Erigeron annuus* (2n=27); A PMC with 27_I_ at M-I. **d** & **e**) *Carduus nutans* (2n=40); PMCs showed multivalents (arrowed) at M-I. **f**) *Geranium pratense* (2n=56); PMC with multivalents (arrowed) at M-I. **g** & **h**) *Taraxacum officinale* (2n=32); PMCs showed multivalents (arrowed) at M-I. **i**) Two PMCs involved in chromatin transfer (arrowed) at P-I. **j**) Two PMCs involved in chromatin transfer (arrowed) at M-I. **k**) Two PMCs involved in chromatin transfer (arrowed) at A-I. **l**) Two PMCs involved in chromatin transfer (arrowed) at T-II. **m**) Group of PMCs involved in cytomixis (arrowed). **n**) Fusion of two monads. **o**) Intermicrosporal chromatin transfer among microspores of three tetrads. **p**) Cytoplasmic channels among microspores of a tetrad forming ring. **q**) Hypoploid (2n=24) and Hyperploid (2n=28) PMCs in *Thymus linearis* (2n=26). **r**) An enucleated PMC. **s**) A PMC at M-I showing pycnotic materials (arrowed). **t**) A PMC at M-I showing chromatin stickiness. **u**) Two PMCs at M-I showing early disjunction of bivalents (arrowed). **v**) A PMC at A-I showing late disjunction of chromosomes (arrowed). **w**) A PMC at A-I showing chromatin bridges (arrowed). **x**) *Dianthus angulatus* (2n=30); A PMC with 30_I_ at M-I. **y**) *Inula cuspidata* (2n=20); A PMC with 20_I_ at M-I

Among the 49 diploid taxa, *Achillea millefolium* L. (*n*=9; 2x) have been noticed for the first time with chromosomal multiple associations which is collected from Chittkul region of Kinnaur district at the altitude of 3450m. In this species chromosomes were involved in the formation of chain, zigzag and ring type quadrivalents (Fig. 1a, b). These multivalents showed delayed segregation as comparative to normal bivalents during anaphases which become the cause of abnormal microspore formation and low pollen fertility.

### Chromosomal multiple association in polyploids

Four polyploids named *Erigeron annuus* (L.) Pers. (2n=27; 3x) (Fig. 1c), *Carduus nutans* L. (2n=40; 4x) (Fig. 1d, e), *Geranium pratense* L. (2n=56; 4x) (Fig. 1f), and *Taraxacum officinale* F. H. Wigg. (2n=32; 4x) (Fig. 1g, h) detected to be imbalanced polyploids having irregular pattern of pairing of chromosomes. The analysis of variable frequency of multivalents and univalents provide the knowledge about the nature of polyploidy. In *Erigeron annuus*, the presence of high frequency of unpaired chromosomes specified the allopolyploid nature of studied taxa. Presence of some multivalents in *Carduus nutans* (4x), *Geranium pratense* (4x) and *Taraxacum officinale* (4x) indicated towards the segmental allopolyploid nature in which 34.83%, 6.47% and 42-52% chromosomes were recorded to be involved in multivalent formation respectively. Resultant of which irregular chromosomes segregation during anaphases, abnormal sporad formation, unequal sized fertile pollen grains and also unstained/sterile pollen grains were observed.

### Cytomixis

The phenomenon of cytomixis involving inter PMCs transfer of chromatin material had been reported to occur in 40 species (Table 2). The chromatin transfer in these species involving varying number of PMCs (2-28) takes place during all the stages of meiosis-I and II (Fig. 1i-m). Interestingly, the cytoplasmic channels showing chromatin transfer are also recorded among microspore units of sporads in *Clematis orientalis* var. *acutifolia*, *Dianthus angulatus* and *Heracleum candicans* (Fig. 1n-p)*.* As a result of partial or complete chromatin transfer between meiocytes, hypo-, hyperploid and enucleated PMCs have been observed in majority of these species (Fig. 1q, r). In the present investigations, nucleolus also gets transferred along with the chromatin material from the donor PMC to the recipient PMC and the resultant PMC are depicted with two nucleoli as observed in *Astragalus grahamianus*, *Salvia nubicola*, *Thalictrum cultratum*, *Trigonella pubescens*, and *Vicia pallida*. All these taxa showed a variety of meiotic abnormalities included fragmentation and pycnosis of chromatin (Fig. 1s), interbivalent connections, irregular segregation of bivalents, aberrant microspores, and pollen sterility and heterogeneous sized fertile pollen grains in connection with this phenomenon of chromatin transfer.

**Table 2:** Consolidated data on cytomixis in the presently studied species

S.No.	Taxa	Meiotic chromosome number (n)	Ploidy level	% age of PMCs with cytomixis	No. of PMCs involved in cytomixis	Meiotic stages	Meiotic course	Pollen fertility %age	Pollen size
1.	*Anemone rivularis*	8	2x	1.28	2	AI/II, TI/II	Abnormal	94	Uniform
2.	*Aquilegia fragrans*	7	2x	4.2	2-3	EPI, MI	Normal	99	Uniform
3.	*Astragalus grahamianus*	8	2x	71.27	2-group	MI-TII	Abnormal	92	Uniform
4.	*A. graveolens*	8	2x	17.02-20.55	2	PI-TI	Abnormal	96-97	Variable
5.	*Calamintha clinopodium*	10	2x	7.20	2	MI, TI	Abnormal	99	Uniform
6.	*Chaerophyllum acuminatum*	11	2x	17.44	2-6	AI-TII	Abnormal	96	Uniform
7.	*Clematis grata*	8	2x	3.68	2-3	AI, TI	Abnormal	88-98	Uniform
8.	*C. graveolens*	8	2x	0.56-66.67	2-28	PI-TII	Abnormal	91-98	Variable
9.	*C. orientalis* var. *acutifolia*	16	4x	9.33-29.80	2-3	EPI-Tetrad	Abnormal	84-94	Variable
10.	*Crepis multicaulis*	5	2x	7.67-11.10	2-10	PI-AI	Abnormal	92-96	Uniform
11.	*Cynoglossum zeylanicum*	12	2x	1.56	2	A-I	Abnormal	92	Uniform
12.	*Dianthus angulatus*	2n=30	2x	14.65	2-4	MI-Tetrad	Abnormal	58-62	Variable
13.	*Eriocycla caespitosa*	11	2x	1.88	2-3	PI-TII	Abnormal	97	Uniform
14.	*Fragaria nubicola*	7	2x	17.45	2-8	MI-AI	Normal	95	Uniform
15.	*Heracleum candicans*	11	2x	26.53	2	EPI-Tetrad	Abnormal	95-96	Variable
16.	*Hippophae rhamnoides*	9	2x	7.95-43.90	2-4	EPI-TI	Abnormal	93-99	Variable
17.	*Indigofera heterantha*	24	6x	21.11-26.00	2-3	EPI-TI	Normal	98-99	Uniform
18.	*Leptorhabdos benthamiana*	7	2x	9.45	2	MI	Abnormal	96	Variable
19.	*Lotus corniculatus*	6	2x	18.34	2-3	PI-TII	Abnormal	88	Uniform
20.	*Medicago falcata*	8	2x	15.43-52.11	2-4	EPI-MI	Abnormal	89-92	Uniform
21.	*Melilotus alba*	8	2x	8.67	2-3	EPI-MI	Abnormal	95-96	Uniform
22.	*Mentha longifolia*	12	2x	2.67	2-5	MI	Normal	99	Uniform
23.	*Nepeta erecta*	9	2x	8.11-14.17	2-group	EPI	Abnormal	92-98	Uniform
24.	*Nicotiana tabacum*	24	4x	22.80	2-3	EPI-MI	Abnormal	87	Uniform
25.	*Pedicularis bicornuta*	8	2x	11.93-31.63	2	MI-TI	Abnormal	96-97	Variable
26.	*Potentilla atrisanguinea* var. *atrisanguinea*	28	8x	38.56	2-5	EPI	Abnormal	94-96	Variable
27.	*Ranunculus laetus*	14	4x	22.51-24.05	2-4	EPI-MI	Abnormal	89-90	Variable
28.	*Salvia nubicola*	8	2x	26.02-30.88	2-4	EPI-MI	Abnormal	96-97	Uniform
29.	*Senecio krascheninnicovi*	10	4x	18.29	2-11	MI-TI	Abnormal	96	Variable
30.	*Silene edgeworthii*	12	2x	13.87	2-3	AI-TII	Abnormal	83	Variable
31.	*Spergularia diandra*	18	4x	2.87	2	MI, TII	Abnormal	92	Uniform
32.	*Thalictrum cultratum*	21	6x	47	2-3	EPI-MI	Abnormal	91-94	Variable
33.	*T. foetidum*	21	6x	1.70-43.20	2-6	EPI-TII	Abnormal	78-98	Variable
34.	*T. minus*	7	2x	2.04	2-3	EPI-MI	Normal	98	Uniform
35.	*Thymus linearis*	13	2x	26.45	2-3	MI-TI	Abnormal	93	Variable
36.	*Trifolium repens*	16	4x	5.34-6.02	2-4	MI, AI	Normal	90-91	Uniform
37.	*Trigonella emodi*	8	2x	15.88-17.24	2-3	EPI-AI	Abnormal	93-94	Variable
38.	*T. pubescens*	8	2x	47.05-62.88	2-group	EPI-TII	Abnormal	38-54	Variable
39.	*Vicia pallida*	12	4x	1.52-2.20	2-3	AI/II, TI	Abnormal	83-89	Variable
40.	*V. rigidula*	12	4x	6.52	2-3	EPI, TI	Abnormal	80-81	Variable

### Chromosome stickiness

The phenomenon of chromosome stickiness causing the chromosome aggulination or sticky appearance of chromosomes reported for the first time in maize [11] and attributed it to a mutation caused by a recessive gene called sticky (*st*). Chromosome stickiness was presently scrutinized more frequently in the PMCs during M-I in *Anemone rivularis* (*n*=8), *Astragalus graveolens* (*n*=8), *Berberis kunwarensis* (*n*=14), *Clematis grata* (*n*=8), *C. graveolens* (*n*=8), *C. orientalis var. acutifolia* (*n*=16), *Cuscuta reflexa* (*n*=16), *Erigeron annuus* (2n=27), *Geranium pratense* (n=28), *Heracleum candicans* (*n*=11), *Hippophae rhamnoides* (*n*=9), *Lotus corniculatus* (*n*=6), *Myosoton aquaticum* (*n*=14), *Nicotiana tabacum* (n=24), *Pedicularis bicornuta* (*n*=8), *Ranunculus laetus* (*n*=14), *R. sceleratus* (*n*=16), *Spergularia diandra* (*n*=18), *Vicia pallida* (*n*=12) and *Vicia rigidula* (*n*=12) (Fig. 1t). In these species, the severe chromatin stickiness enhanced the formation of pycnotic nuclei and delayed separation of bivalents at A-I/II, laggards and chromatin bridges, micronuclei, and ultimately resulted into sterile pollen grains.

### Nonsynchronous disjunction

Nonsynchronous disjunction of bivalents (early and late) is either found in hybrid taxa or the species having different sized chromosomes and rate of chiasma terminalization [12, 13] or associated with other meiotic abnormalities [14]. Some of the presently studied species showed either precocious disjunction (*Ranunculus laetus*, *n*=14; *Impatiens brachycentra*, *n*=7; *Chaerophyllum villosum*, *n*=11; *Aster molliusculus*, *n*=9; *Cosmos bipinnatus*, *n*=12; *Crepis multicaulis*, *n*=5; *Salvia nubicola*, *n*=8) (Fig. 1u) or late disjunction (*Clematis graveolens*, *n*=8; *C. orientalis* var. *acutifolia*, *n*=16; *Delphinium roylei*, *n*=8; *Papaver dubium*, *n*=14; *Trigonella pubescens*, *n*=8; *Vicia pallida*, *n*=12; *V*. *rigidula*, *n*=12; *V. sativa*, *n*=6; *V. tenera*, *n*=7; *Anaphalis virgata*, *n*=14; *Crepis sancta*, *n*=5; *Erigeron acer*, *n*=9; *Inula cuspidata*, *n*=10; *Lactuca orientalis*, *n*=9; *Youngia glauca*, *n*=8; *Cuscuta reflexa*, *n*=16; *Physalis minima*, *n*=24; *Leptorhabdos benthamiana*, *n*=7; *Pedicularis bicornuta*, *n*=8; *Hippophae rhamnoides*, *n*=9) (Fig. 1v, w) of some bivalents. On the other hand, *Ranunculus sceleratus*, *n*=16; *Thalictrum cultratum*, *n*=21; *Berberis kunwarensis*, *n*=14; *Inula cappa*, *n*=10; and *Senecio krascheninnicovi*, *n*=10 showed both early and late disjunction of 1-2 bivalents.

Early disjunction of bivalents normally does not affect the normal distribution of chromosomes at A-I. While the late disjuction of bivalents in *Ranunculus sceleratus*, *Papaver dubium*, *Vicia rigidula*, *V. sativa*, *Erigeron acer*, *Inula cappa*, *I. cuspidata*, *Lactuca orientalis*, *Youngia glauca*, *Cuscuta reflexa* and *Physalis minima* causes lagging of chromosomes, chromatin bridges and consequently reduced pollen fertility [4, 15, 16].

In *Delphinium roylei*, *Vicia pallida*, *V. sativa*, *Inula cappa*, *I. cuspidata*, *Cuscuta reflexa*, *Lactuca orientalis* and *Youngia glauca*, larger sized bivalents scrutinized with decelerated segregation at A-I/II. In *Delphinium roylei*, *Vicia sativa*, and *Cuscuta reflexa*, this abnormality does not seem to affect the pollen fertility but in *Vicia pallida*, *Inula cappa*, *I. cuspidata*, *Lactuca orientalis* and *Youngia glauca*, late disjunction have resulted in the presence of laggards which may originate micronuclei at telophase-I/II and leading to reduced pollen fertility.

### Asynaptic mutants

The lack of chromosome pairing and inability to generate or retain chiasmata during P-I led to asynaptic and desynaptic mutant formation. In *Dianthus angulatus* (2n=30) (Fig. 1x) and *Inula cuspidata* (2n=20) (Fig. 1y), chromosomes remain unpaired as univalents which disorganized into more than standardized number of chromatin poles during segregation of chromosomes at A-I/II. A high frequency of aberrant microspores including monads, dyads, triads, tetrads with micronuclei and polyads, sterile gametes and heterogeneous sized pollen grains are the major consequences.

### Syncyte PMCs

**Fig. 2:**
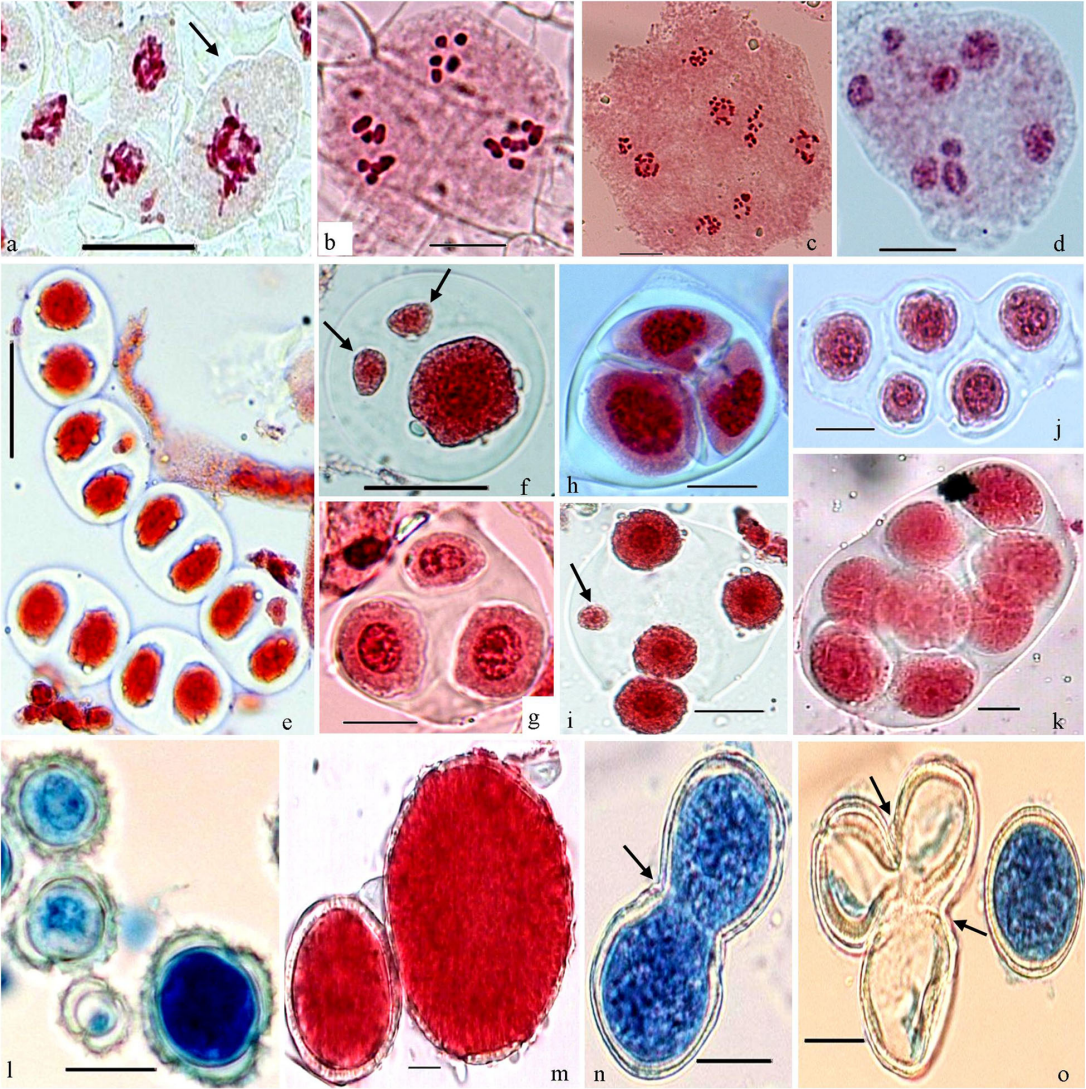
Various Meiotic deformities during meiosis. **a**) *Clematis graveolens* (2n=16); a syncyte PMC (arrowed). **b**) *Inula cuspidata* (2n=20); chromosomes disorganized into unequal sized 3 groups at A-I. **c**) *Geranium pratense* (2n=56); chromosomes disorganized into unequal sized 8 groups at A-II. **d**) *Dianthus angulatus* (2n=30); chromosomes disorganized into unequal sized 8 groups at T-II. **e**) A group of dyads with and without micronuclei. **f**) A monad with two micronuclei. **g** & **h**) Triad. **i**) A tetrad with one micronucleus (arrowed). **j**) A pentard. **k**) A polyad. **l**) Fertile and Sterile pollen grains. **m**) Apparently fertile heterogeneous sized pollen grains. **n**) Fused fertile pollen grains. **o**) Fused sterile pollen grains

The fusion of two or more meiocytes presently scrutinized in *Clematis graveolens* (2n=16) (Fig. 2a) and *Dianthus angulatus* (2n=30) during the early stages of meiosis-I. However the occurrence of these synmeiocyte was found to be at very low frequency. These PMCs with double content of genome undergo the meiosis and led to the formation of polyploid gametes. In both cases, the male gametes are larger in size and well fertile and able to fertilize the female gamete and might play significant role in the origin of intraspecific polyploids.

### Abnormal spindle

Spindle organization were found to be abnormal during meiosis in *Colutea nepalensis* (2n=16), *Dianthus angulatus* (2n=30), *Erigeron annuus* (2n=27), *Geranium pratense* (2n=56), *Inula cuspidata* (2n=20), *Papaver dubium* (2n=28) and *Taraxacum officinale* (2n=32). Here, the chromosomes fail to assemble on the spindle fibres during metaphases which is the essential property for their synchronous segregation, so they finally congregate into disparate sized chromatin nuclei at anaphases and telophases (Fig. 2b-d). As a consequence, abnormal microspores (Fig. 2e-k) are resulted which lead to low pollen fertility (Fig. 2l) and pollen grains of heterogeneous sizes (Fig. 2m).

### Fused Pollen grains

Fusion among 2-3 pollen grains resulting into large sized pollen grains have been observed in *Clematis graveolens* (*n*=8) (Fig. 2n, o). In this species, 2-3 pollen grains are fused by forming cytoplasmic channels.

## Discussion

Analysis of meiotic chromosome associations in diploid and polyploid plants can give precise knowledge of chromosome homology and synapsis during meiosis [17]. Inter/intraspecific hybrid origin of a plant might contemplate the level of chromosome homology. Structural heterozygosity for reciprocal translocation arguably denote the configuration of typically ring, zigzags and chains of four chromosomes in *Achillea millefolium* (*n*=9; 2x)*.* Further, persistence of multivalents is also determined by the presence of homologous gene sequences and keep possession of chiasma in multiple associated chromosomes. Occurrence of univalents and multivalents in complex and instable polyploid genome of *Erigeron annuus* (2n=27; 3x), *Carduus nutans* (2n=40; 4x), *Geranium pratense* (2n=56; 4x), and *Taraxacum officinale* (2n=32; 4x) with distorted behaviour during gamete formation led to low reproductive success. Such chromosomal associations occur in nature at a low rate, may arise spontaneously or induced by a variety of factors including chemical or irradiation treatments [18]. The inverse correlation between frequency of chromosome associations and pollen fertility also reported in *Hyoscyamus muticus* [19] and *Brassica campestris* var. *toria* [20].

Cytomixis, an evolutionary, panoramic, cytological phenomenon which is a possible cause of aneuploidy and polyploidy in species [7], and produce unreduced pollen grains as reported in *Hordeum* species [21], *Dianthus angulatus* [22], and *Houttuynia cordata* [23] *Chlorophytum borivilianum* [24]. Many workers have pointed of view that the reduction in pollen viability is due to cytomixis [23, 24]. Till date, there is no clear cut opinion regarding the origin and nature of the cytomixis. Some interpretation which are thought to be responsible for cytomixis include the action of chemical agents such as colchicines [25], the use of herbicides [26], physiological and environmental factors [27], stress factors and genetic control [28]. In presently studied taxa, genetic factors and pressure of stress environment conditions seems to be the originator of this phenomenon of cytomixis [28–30].

Chromosome stickiness co-occurred with cytomixis appear to be commenced the pycnosis and degeneration of chromatin material as earlier reported by other workers [22, 31–33]. Several workers have reported that chromosome stickiness may be either under genetic control [34] or due to improper folding of chromosome fibres [35], or may also have been caused by other factors such as X-rays [36] and low temperature [37]. The presence of aluminum in the soil, besides the genetic factor, may have also caused chromosome stickiness in maize [32].

It becomes necessary for all the chromosomes to get synchronously disjucted during anaphases for the genome stability in the species. Expeditious rate of chiasma terminalization and least genic homology caused early disjunction of bivalents. Delayed segregation in *Delphinium roylei*, *Vicia pallida*, *V. sativa*, *Inula cappa*, *I. cuspidata*, *Cuscuta reflexa*, *Lactuca orientalis* and *Youngia glauca* during anaphases was due to having large sized bivalents which has also been reported in *Cyathocline purpurea* and *Blumea* spp. [38]. High chiasma frequency and their slow terminalization in large sized chromosomes might be the reason for delayed segregation.

A crucial phenomenon, synapsis, during early prophase stages is essential for gene recombination and evolutionary traits in the taxa [5]. Synaptic mutant might be due to mutations in genes controlling the chromosome pairing process and inability to generate or retain chiasmata between homologous chromosomes [5]. Several researchers have been reported for its spontaneous origin [39] and influenced by many factors like temperature, humidity and chemicals [5]. Presently the individuals of *Dianthus angulatus* and *Inula cuspidata* were growing under same climatic conditions detected with and without synaptic mutation so this make seem likely that a particular another factor might be involved. A concept of interspecific origin or non homology between two genomes could be the reason for synaptic mutation among the presently investigated taxa. A high frequency of unpaired elements in the genome of these taxa led to sterile and 2n fertile gamete formation in the end of meiosis as has also been reported in higher plants [39–41].

The spindle apparatus is normally bipolar and playing a crucial role in the accurate segregation of chromosomes during mitosis and meiosis [42]. Several mutants are known to cause failure of the spindle or impair disjunction mechanisms, like *dv*, *ms28* and *ms43* mutants as reported in maize [43]. *Multipolar Spindle 1* (*MPS1*), a plant-specific protein which is involved in spindle organization in meiocytes has been identified in *Arabidopsis thaliana* [44]. Improperly aligned chromosomes on distorted spindle face either unequal or total failure of segregation during anaphases which might to be taken to lead into restitution nuclei or polyploidy or aneuploidy in resultant gametes.

Also this low temperature stress conditions prevailing in the presently investigated area might lead the amalgamation of PMCs and pollen grains. These syncyte PMCs have earlier recorded in *Brachiaria jubata* [45], *Chrysanthemum* [46], and *Lindelofia longifolia* var. *falconeri* [47]. The fusion of cell wall of pollen grains has also been recorded in intergeneric hybrids [48, 49]*.* The origin of intraspecific polyploidy in these taxa cannot be changed in spite of the low frequency of such fused PMCs and pollen grains.

## Conclusions

Production of unreduced 2n gametes (double sized pollen grains) are the major consequence of these meiotic abnormalities in these studied taxa. Further these 2n male gametes fertilize the female gamete of respective species and led to origin of polyploidy. These all presently detected in the plants growing under the natural conditions particularly freezing temperature prevailing in the area which leads to low sexual reproductive success. The adoption of vegetative mode of reproduction may be the better regeneration method in such plants.

## Methods

### Collection and submission of samples

Study materials were collected from Kinnaur district of Himachal Pradesh (India) during the months of April to September for five years (2007-2011).Voucher specimens of the cytologically worked out individuals were deposited in the Herbarium, Department of Botany, Punjabi University, Patiala (PUN).

### Cytological Analysis and Photomicrographs

For cytological study, young floral buds of dicot plants were fixed in carnoy’s fixative (6 ethanol: 3 chloroform: 1 acetic acid) and then stored at 4°C in 70% alcohol. Standard squash method (1% acetocarmine) was applied for observing all the meiotic stages clearly. Meiocytes, sporads and pollen grains were photomicrographed from the freshly prepared slides using Leica Qwin Digital Imaging System and Nikon Eclipse 80*i* microscope at laboratories situated in Department of Botany, Punjabi University, Patiala.

## Abbreviations

A-I/II: Anaphase-I/II
M-I/II: Metaphase-I/II
P-I/II: Prophase-I/II
PMC: Pollen Mother Cell
T-I/II: Telophase-I/II
